# Effect of Zein‐Persian Gum Water‐in‐Oleogels on Quality Characteristics of Unsaturated Fatty Acid‐Rich and Low‐Fat Croissant

**DOI:** 10.1002/fsn3.4649

**Published:** 2025-03-12

**Authors:** Atefeh Esmaeilinezhad, Hajar Abbasi

**Affiliations:** ^1^ Department of Food Science and Technology, College of Agriculture and Natural Resources, Isfahan (Khorasgan) Branch Islamic Azad University Isfahan Iran

**Keywords:** bigels, croissant, differential scanning calorimetry, fatty acid profile, rheology

## Abstract

Water‐in‐oleogels are structured oils formed using gelators with varying gelation performance. In this study, the production of novel bigels and their consumption as a commercial shortening substitute in croissant formulation to reduce the fat and saturated fatty acid content and produce a healthier product are of concern. Water‐in‐oleogels of sunflower oil were prepared by consuming different combinations of gelators, monoglyceride, Zein protein (2.5%, 5%, and 7.5%), and Persian gum (0%, 1.5%). After assessing their thermal and rheological properties, they were substituted with shortening in the formulation of croissants to reduce fat and saturated fatty acid content. The qualitative properties of the products were assessed from different perspectives. Water‐in‐oleogels had solid, viscoelastic gel‐like structure, with pseudoplastic behavior and an increase in the Zein concentration in the presence of hydrocolloids enhanced the viscoelastic properties and melting enthalpy of created crystals in the bigel structure. The moisture content and hardness of croissants produced with water‐in‐oleogels were higher and lower, respectively, than those in the control. Using hydrocolloid next to increasing the Zein concentration decreased the oil release of the product. The croissant prepared using water‐in‐oleogel containing 2.5% Zein had the lowest density compared to the other samples. The intensity of yellowness, chroma, and browning index were higher in samples produced with water‐in‐oleogels containing low levels of gelators. The water‐in‐oleogel prepared with 2.5% Zein was useful in the production of croissants with favorable quality properties and 15% lower fat compared to the control without significant difference in terms of peroxide value. The total volume of the saturated and essential unsaturated fatty acids in the developed product decreased by 47% and increased by 65%, respectively, next to maintaining the aroma, taste, color, and most of the organoleptic properties.

## Graphical abstract

The moisture content and hardness of croissants produced with water‐in‐oleogels were higher and lower, respectively, than those in the control. Using hydrocolloid next to increasing the Zein concentration decreased the oil release of the product. The water‐in‐oleogel prepared with 2.5% Zein was useful in the production of croissants with favorable quality properties and 15% lower fat compared to the control without significant difference in terms of peroxide value. The total volume of the saturated and essential unsaturated fatty acids in the developed product decreased by 47% and increased by 65%, respectively, next to maintaining the aroma, taste, color, and most of the organoleptic properties.

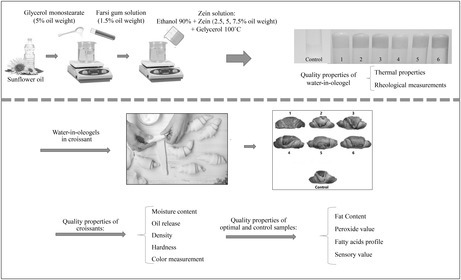

## 1 Introduction

Confectionery and some types of bakery products are delicious, fatty products and good candidates for reducing fat and saturated or *trans*‐fatty acids reduction. Their improved nutritional properties would contribute to the consumers' health (Oh ImKyung and Lee SuYong 2018). Hydrogenated shortenings are usually consumed in the bakery and confectionery because they highly contribute to the quality properties of products with a high oxidative stability index and long‐term shelf life, next to creating satisfactory texture, flavor, and aroma (Macias‐Rodriguez and Marangoni 2018). The excessive consumption of saturated fatty acids increases undesirable cholesterol, obesity, and the risk of cardiovascular diseases (Anushree et al. 2017). Vegetable oils with high content of unsaturated fatty acids lack the physical properties required for this industry (Meng et al. 2019).

Organogelation is a new method to form a gel‐like structure in edible liquid oil, where the gelator is the agent to connect fatty acid chain and leads to the formation of a three‐dimensional network capable of stabilizing the liquid phase (Hwang et al. 2018). Researchers (Wijarnprecha et al. 2018; Chen and Zhang 2020) assessed the synergistic effect of gelator types with different gelation efficiency, like monoglycerides, alcohols or fatty acid esters, phospholipids, and phytosterols, together with different waxes or protein–polysaccharide, to yield the appropriate texture and stability of the product even at low concentrations, without increasing the content of saturated and *trans*‐fatty acids. The assessments run on oleogels prepared with different concentrations of hydroxypropyl methyl cellulose, sodium caseinate, and beeswax have revealed that the concentrations of hydroxypropyl methyl cellulose and sodium caseinate, due to the formation of a complex and strong network, are more influenced factors on oil binding capacity, textural properties, and rheology of oleogels. A considerable improvement in oil binding capacity and restoration of the sample structure was observed by adding beeswax (Alizadeh et al. 2020). The effect of bigels, including five gelators, hydroxypropyl methylcellulose, monoacylglycerol, sodium stearyl lactate, rice bran wax, and beeswax consumption on cookies was assessed and the results revealed that the cookie baked with oleogel containing monoacyl glycerol and rice bran wax had a homogeneous porous structure, proper color, ideal crispy texture, and proper rheology properties, and in this respect, it was similar to the sample prepared with shortening. The effect of two different oleogels prepared with high oleic soybean oil, monoacylglycerol, and rice bran wax in bread and cracker formulas was assessed in comparison to shortening, and the results revealed that this change in oil types did not significantly change the dough properties. Oleogel based on high oleic soybean oil with monoacylglycerol has a high softening effect on bread, less hardness on crackers, and a high potential to replace shortening without reducing the quality of dough and final bakery products (Zhao, Rao, and Chen 2022). There exists a strong dependency of the quality properties of the product on the type and concentration of gelators consumed in their organogel (Li et al. 2021).

Water‐in‐oleogels are oil‐in‐water emulsions that keep water in the oleogel structure and can reduce the fat content in food products. The production of these oleogel types and their consumption in food products is the focus of researchers. In this context, the researchers first produced watery and water‐free oleogels containing different percentages of oil types and carnauba wax and next assessed the effect of their consumption on the properties of sponge cake, where they found that these organogels had no adverse effect on the quality of batter or textural and sensory properties of products. Consuming oleogels, which are rich in unsaturated fatty acids, in cake formulations is proper for producing a healthier product, with low calories and the same quality as the control sample (Pehlivanoglu et al. 2018). The development of low‐fat chocolate spreads by consuming water emulsions in oleogel containing corn oil‐glycerol monostearate, where the physical, rheological, and sensory properties of the sample were analyzed and compared, is another finding by Tirgarian et al. (2023), where the water emulsion made of a 45:55 water to oleogel ratio (45% replacement of oleogel with water) consumed in the production of chocolate with integrated microstructure, proper viscoelastic properties, and spreadability similar to the control sample was evident. Proteins as the most important biopolymers able to form foam and stable water‐in‐oil emulsions and polysaccharides as other food biopolymers that contribute to the formation of gel‐like complexes, can be consumed in the production of water‐in‐oleogels (Ruiz‐Capillas et al. 2013; Wang et al. 2023). Zein is an edible hydrophobic protein yield from corn prolamin, which is generally consumed as a coating in the food and pharmaceutical industries (Nephomnyshy, Rosen‐Kligvasser, and Davidovich‐Pinhas 2020). Zein is consumed in the food and pharmaceutical industries, such as the production and stabilization of emulsions, foams, and capsules (Corradini et al. 2014). Researchers prepared a series of oleogels stabilized with Zein and glycerol, and by assessing the rheological and microstructural properties of oleogels as a function of glycerol and Zein concentrations, they revealed that by adding more Zein and glycerol concentrations, oleogels became denser and led to the improvement of rheological properties and oil storage capacity (Keshanidokht et al. 2023). Persian gum (Zedo) is a natural polysaccharide obtained from Iranian mountain almond trees and is consumed in the food industry to increase viscosity, improve texture, stabilize foam and emulsion, and form film and coating (Tamimi, Rajabi, and Pezeshki‐Modaress 2020). In this study, the combination of Persian gum and Zein protein was assessed as a gelator in the production of water‐in‐oleogel.

According to Espert et al. (2023), products baked from pastry dough, like croissants, belong to the categories of bakery products, which have a unique structure of fat layers and thin laminated dough. These features provide a light, delicate, and desirable flaky texture. In this study, the production of water‐in‐oleogels using varying ratios of Zein protein and Persian gum, in the presence of glycerol monostearate, was evaluated as a substitute for commercial shortening in croissant formulations. This approach aims to reduce fat and saturated fatty acid content while maintaining the desired quality properties of the final product.

## 2 Materials and Methods

### 2.1 Materials

Sunflower oil was purchased from a local supermarket; Persian gum was purchased from a Medicinal Plants Research Center (Isfahan‐Iran); food‐grade Zein from corn (89.5%) was purchased from Titrachem (Canada); GMS (99.5%) was purchased from Alpha Aesar (USA); glycerol (98%) and edible ethanol (96%) were purchased from Bio‐Lab Chemicals (Iran); flour and commercial shortening were purchased from Ghoncheh company (Iran); yeast and flour improver were purchased from Golnan‐Ceratus company (Belgic‐Iran), salt, sugar, and eggs were purchased from a local supermarket.

### 2.2 Preparation of Water‐in‐Oleogels

Zein was dissolved in 90% (w/w) aqueous ethanol, and next, food‐grade glycerol was added to this solution at 100°C. Sunflower oil and GMS were heated at 60°C for 5 min. The Persian gum was dissolved in water, and the solution was added to the oil phase while being heated and then emulsified. This emulsion was gradually added to the Zein‐ethanol‐glycerol mixture while being stirred at 500 rpm for 1 min. The liquid phase contained 60% oil and 40% water‐alcohol (9% ethanol, 20% glycerol, and 11% water). Under continuous stirring at 70°C, the ethanol evaporated and the protein denatured. The yield oleogels were quickly stored at 4°C for 24 h before being homogenized at 25°C at 10,000 rpm for 1 min (Nephomnyshy, Rosen‐Kligvasser, and Davidovich‐Pinhas 2020). The volumes of the produced water‐in‐oleogel components are tabulated in Table 1.

**TABLE 1:** Components of the water‐in‐oleogels.

Samples	Oil (%)	GMS (%)	Zein (%)	Persian gum (%)
Control	60	5.0	0	0
1	60	5.0	2.5	0
2	60	5.0	5.0	0
3	60	5.0	7.5	0
4	60	5.0	2.5	1.5
5	60	5.0	5.0	1.5
6	60	5.0	7.5	1.5

### 2.3 Thermal Properties of Water‐in‐Oleogel

The thermal properties of oleogel samples were assessed through a calorimeter (DSC131 EVO Setaram, France). For this purpose, about 10 mg of the sample was weighed in an aluminum container with a lid. The samples were heated from 5°C to 120°C at a 5°C/min ratio (Silva et al. 2021).

### 2.4 Rheological Measurements of Water‐in‐Oleogel

The rheological properties of oleogels were measured by running the rotational test through a Rheometer 302 (Anton Paar, Austria) attached to a temperature control system. Tests were run on a 40 mm parallel plate with a 1000 μm gap (Meng et al. 2019).

### 2.5 Preparation of Croissants

**FIGURE 1:**
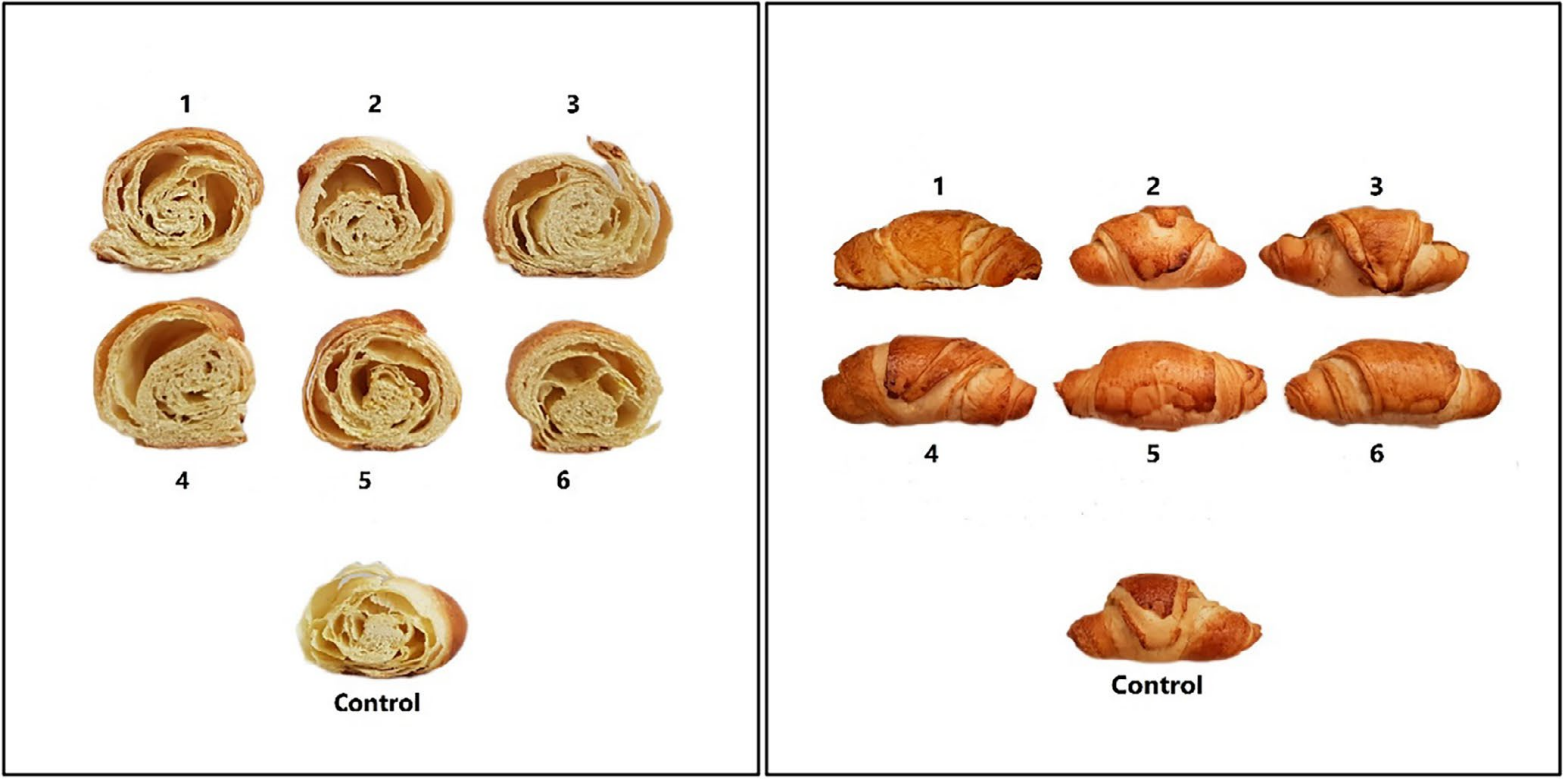
Croissant samples prepared in this research. Control is the croissant prepared with shortening. Croissants prepared with water‐in‐oleogel contain 1: (2.5% Zein), 2: (5% Zein), 3: (7.5% Zein), 4: (2.5% Zein and 1.5% Persian gum), 5: (5% Zein and 1.5% Persian gum), 6: (7.5% Zein and 1.5% Persian gum).

To prepare croissants, all ingredients, Table 2, were poured into the blender (SBG 5730, SUNNY, Iran) and mixed for 20 min. Water was added gradually during mixing. The yield was seven pieces of dough were placed in the spreader to become layers. Water‐in‐oleogel was layered upon each piece of dough, rolled, and placed inside the fermentation rooms for 30 min, followed by baking in the oven at 140°C for 30 min. The image of produced croissants is shown in Figure 1.

**TABLE 2:** Raw materials for baking croissants.

	Materials ingredients	The volume of materials (g)
1	Flour	1000
2	Yeast	70
3	Flour improver	6
4	Salt	6
5	Sugar	200
6	Shortening	50
7	Egg	35
8	Vanilla	2

### 2.6 Moisture

The moisture content of croissant samples was measured on the first and tenth days of storage. Five grams of each sample was placed in the constant weight aluminum container and put in the oven at 105°C for 60 min. After cooling in a desiccator, it was reweighed, and the moisture content of the product was calculated through the following equation (Mert and Demirkesen 2016):

(1) $$\text{Moisture}\;\left(\%\right)=\left(W_{2}-W_{3}\right)/\left(W_{2}-W_{1}\right)\times 100$$

where *W*_1_ is the container weight, *W*_2_ is the weight of the container containing the sample before heating, and *W*_3_ is the weight of the container containing the sample after heating.

### 2.7 Oil Release

Two filter papers (36 cm^2^) were weighed, and 10 g of the sample was placed between them; next, a force weighing 1 kg was placed on the sample and the filter paper for 2 h. The weight of the filter paper and the released oil from the sample was measured and the percentage of oil released from the sample was calculated (Abbasi and Pourdavoud 2020).

### 2.8 Hardness

The hardness of the samples was measured through a texture analyzer (STM‐20, Santam, Iran) on the day after production and five and 10 days later. For this purpose, the samples were cut into rectangular cube pieces of 2 × 2.5 × 2.5 cm and were compressed to 40% of the initial height by a circular probe with a diameter of 5 cm and a speed of 60 mm/s. The force required to compress the samples was reported as the hardness (Espert et al. 2023; Karimi and Abbasi 2023).

### 2.9 Density

Measuring the volume and apparent density of the samples was determined by calculating the ratio of croissant weight to volume ratio by applying the technique of rapeseed displacement (Tang and Ghosh 2021).

### 2.10 Colorimetric

The color of the crust and the crumb of the croissant samples was measured through an Ultra Scan PRO colorimeter (Hunter Associates Laboratory Inc., Reston, VA, USA), and three indices L* (darkness/lightness), a* [greenness (−)/redness (+)], and b* [blueness (−)/yellowness (+)], were evaluated (Azmoon et al. 2021; Mirhosseini and Abbasi 2021). Chroma (C*), and Brown Index (BI) were calculated through the following equations:

(2) $$C^{*}=\sqrt{\left(a^{2}+b^{2}\right)}$$

(3) $$\mathrm{BI}=\frac{100\times \left(x-0.31\right)}{0.172}$$

(4) $$x=\frac{a+1.75L}{5.645L+a-3.012b}$$

### 2.11 Fat Content

Five grams of sample in the thimble was placed in Soxhlet (Soxhlet extractor, PECOFOD, Iran), and the fat extraction was made in 6 h with hexane. Hexane was removed at 45°C by a rotary device, and the remaining fat was weighed. The percentage of fat was calculated through the following equation (Fayaz, Goli, and Kadivar 2017):

(5) $$\mathrm{Fat}\;\left(\%\right)=\left(M_{1}–M_{2}\right)/M\times 100$$

where *M* is the weight of the initial sample, *M*_1_ is the weight of the Soxhlet flask and the remaining fat, and *M*_2_ is the weight of the Soxhlet flask.

### 2.12 Peroxide Value

Five grams of fat extracted by the Soxhlet method were weighed and stored in an Erlenmeyer flask with 30 mL of 2:3 acetic acid‐chloroform solutions and 0.5 mL of saturated potassium iodide solution for 1 min in the dark. After 1 min, 30 mL of distilled water and 0.5 mL of 0.01 starch indicator were added to the sample to assure the appearance of a blue color in the presence of peroxide in the sample. Then titration was done with 0.01 normal sodium thiosulfate until the blue color faded away, and the peroxide value was calculated through the following equation (Silva et al. 2021):

(6) $$\mathrm{PV}=\left[\left(V_{s}-V_{b}\right)\times N\times 1000\right]/W$$

where *V*_s_ is the volume of thiosulfate consumed by the sample, *V*_b_ is the volume of thiosulfate consumed by the control, *N* is the normality of sodium thiosulfate, and *W* is the sample mass.

### 2.13 Profile and Fatty Acid Volume

The fatty acid composition of the tested samples was evaluated using gas chromatography (GC–MS/MS, Agilent 7890A, USA). Fatty acid methyl esters were prepared by methoxylation with sodium methoxide (N 0.5). The carrier gas was hydrogen with a 1.1 mL/min flow rate. A flame enzymation detector was applied, and the sample injection volume was 1 μL (Banaeifar and Abbasi 2023).

### 2.14 Sensory Evaluation

To evaluate the sensory elements of croissant samples, 20 trained panelists were used. The relevant forms of considered traits and scores were provided to the evaluators for comparison and scoring texture, taste, aroma, color, and overall acceptance (Azaripour and Abbasi 2019).

### 2.15 Statistical Analysis of the Data

The Zein protein at 2.5%, 5%, and 7.5% oil weight concentrations with and without 1.5% Persian gum in the presence of 5% GMS were consumed as gelators to produce water‐in‐oleogels of sunflower oil. Qualitative properties of six water‐in‐oleogels and formulated croissants with them were measured in triplicate. Statistical analysis was run through (ANOVA) according to a completely randomized design followed by LSD's test to compare the means of the dependent variables at a significant probability level through the statistical software version 9 (SAS Institute, USA). Results are presented as the mean ± SD. Diagrams were drawn by applying the Microsoft Excel 2016 software.

## 3 Results and Discussion

### 3.1 Thermal Properties of Water‐in‐Oleogel

**FIGURE 2:**
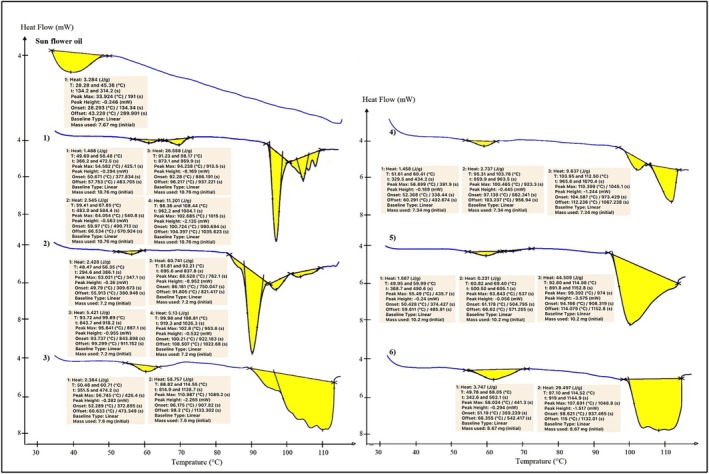
Thermal properties of water‐in‐oleogels. Samples contain 1: (2.5% Zein), 2: (5% Zein), 3: (7.5% Zein), 4: (2.5% Zein and 1.5% Persian gum), 5: (5% Zein and 1.5% Persian gum), 6: (7.5% Zein and 1.5% Persian gum).

Determining the thermal properties of the oil and fat types is important to control their spreadability and stability in different storage and processing conditions. The melting point of fat is influenced by its chain length and saturation degree of fatty acids (Abdolmaleki et al. 2022). Figure 2 shows that the thermal properties of oleogels, like the melting point and melting enthalpy, were obtained based on the endothermic peaks from differential scanning calorimetry (DSC). The control had only one melting point at 43.23°C, while the water‐in‐oleogels revealed 2–4 different peaks at different temperatures, indicating the presence of different melting behaviors of the crystals. The oleogels containing 2.5% and 5% Zein with multiple endothermic peaks at different temperatures had lower melting speed and greater heat resistance than others. The presence of Persian gum slightly increases the melting point of water‐in‐oleogel, and also the interaction of Zein–Persian gum decreased the melting enthalpy of the samples at all Zein concentrations. Thermal stability is a critical parameter where the maintenance of textural properties is essential in product quality (Pang et al. 2020; Wang et al. 2024). Higher thermal stability and melting point of oleogels produced with high concentrations of hydrocolloid containing sodium caseinate, guar gum, and xanthan gum were reported in the other study (Abdolmaleki et al. 2022).

### 3.2 Rheological Measurements

**FIGURE 3:**
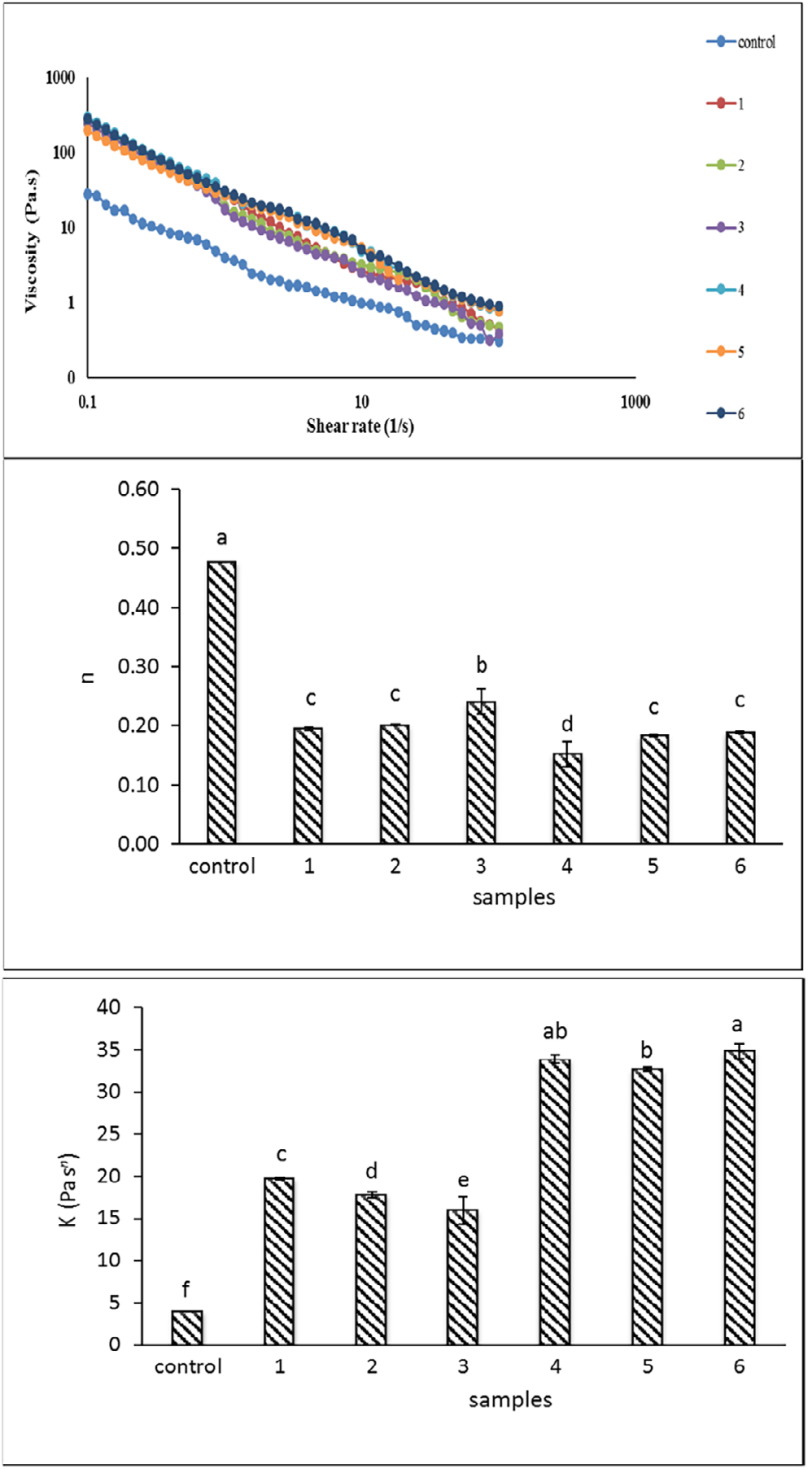
Viscosity of water‐in‐oleogels at different shear rates; Mean comparison of flow behavior index (*n*), and consistency coefficient (*K*) according to the power law model. Control: (without Zein and Persian gum), 1: (2.5% Zein), 2: (5% Zein), 3: (7.5% Zein), 4: (2.5% Zein and 1.5% Persian gum), 5: (5% Zein and 1.5% Persian gum), 6: (7.5% Zein and 1.5% Persian gum).

Unlike sunflower oil, which has a Newtonian behavior, the viscosity of all samples assessed in this study decreased with an increase in shear rate, indicating shear thinning behavior (Figure 3a). The formation of fat crystals and their changes due to stress is the main reason for observing shear thinning behavior in water‐in‐oleogels (Figure 3b). Shear thinning behavior was observed in oleogel prepared of canola oil with xanthan, pea protein concentrates, and isolate (Mohanan, Nickerson, and Ghosh 2020). Changes in the consistency coefficient have been reported in studies run on organogels. An increase in γ‐oryzanol and β‐sitosterol content as a gelator consumed in coconut oil increased the consistency coefficient of samples. This phenomenon is due to the more interactions of the oil and the gelator and formation of crystals toward increasing the gelator concentration correspond with enhancing viscosity (Jiang et al. 2019). The power law model was fitted to the data obtained from the rotational rheology test, where two parameters of consistency coefficient and flow index were obtained. The control consistency coefficient is lower than the rest of the samples (*p* < 0.05) and the presence of hydrocolloids caused an increase in the consistency coefficient of the oleogels (Figure 3c). Viscosity increase with increasing gelator concentration in oleogels prepared with egg white protein–xanthan gum has been reported (Jaberi et al. 2020). Water‐in‐oleogels have higher viscosity at a low shear rate compared to the control, but their viscosity reduction rate with an increase in the shear rate was more than the control. The flow behavior index of most water‐in‐oleogels with different concentrations of gelator did not differ considerably from each other, although the increase in protein concentration was effective in increasing this parameter, and this factor was lower in all samples than the control (*p* < 0.05). The effect of protein concentration on improving the viscoelastic properties of Zein‐based oleogels was reported in Tsung, Ilavsky, and Padua (2020) research.

### 3.3 Qualitative Properties of Croissants

#### 3.3.1 Moisture

**FIGURE 4:**
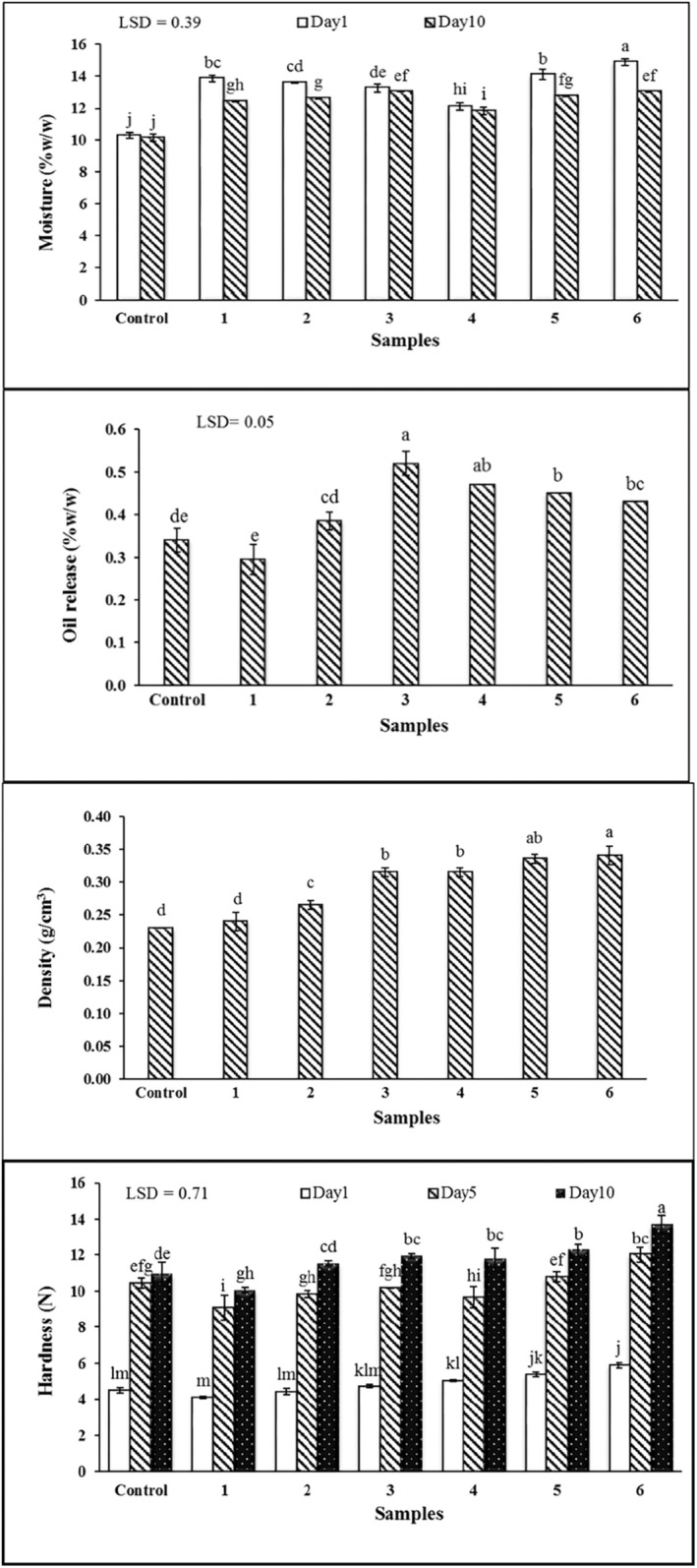
Qualitative properties of croissants. Control is the croissant prepared with shortening. Croissant prepared with water‐in‐oleogel contain 1: (2.5% Zein), 2: (5% Zein), 3: (7.5% Zein), 4: (2.5% Zein and 1.5% Persian gum), 5: (5% Zein and 1.5% Persian gum), 6: (7.5% Zein and 1.5% Persian gum).

Moisture content is one of the important quality indicators in confectionery products that affects their staleness rate (Kim et al. 2017). Moisture reduction is related to its migrating and evaporative during the storage period (Kim et al. 2017). The moisture content assessment of croissants at the beginning and end of the storage period reveals that the samples prepared with water‐in‐oleogel have higher moisture content compared to samples prepared with shortening (Figure 4a). This observation is related to the ability of oleogels to absorb and entrap water molecules and prevent their migration from starch (Mert and Demirkesen 2016). The presence of Persian gum in oleogels with Zein concentrations of 5% and 7.5% is effective in increasing and maintaining the moisture content of the croissants containing water‐in‐oleogel. Hydrocolloids, due to their high power in absorbing and maintaining the moisture during baking and storage, are influential in maintaining product moisture (Kim et al. 2017). In replacing commercial shortening with oleogel in the cookie, it is reported that consuming oleogel with beeswax leads to a decrease in moisture in this product, while consuming oleogel with monoglycerides leads to an increase in the same (Yılmaz and Öğütcü 2015).

#### 3.3.2 Croissant Oil Release

In terms of the volume of oil released from baked products shown in Figure 4b, the samples prepared with water‐in‐oleogel containing 2.5% and 5% Zein did not show any significant difference from the samples prepared with shortening. The addition of hydrocolloids, next to increasing the Zein concentration in the water‐in‐oleogel, led to a decrease in the oil release percentage from croissants. The stability of oleogel and emulsion systems containing oleogel is due to water droplets' entrapment inside the crystal structure and the accumulation of small crystals at the water–oil interface, which prevent oil migration from emulsion structure by forming an ester barrier. Consumption of sunflower oil oleogel containing monoglyceride as a fat substitute in muffin was effective in reducing the volume of oil released from this product (Giacomozzi, Carrín, and Palla 2023). Consumption of shellac in the production of rapeseed oil oleogel protected texture and prevented two‐phase separation during 4 months of storage (Patel et al. 2014). Changing the batter viscosity in the presence of hydrocolloids improves oil retention by creating a coherent and stable structure in the croissant texture network.

#### 3.3.3 Density

Fat crystals contribute to baking products' aeration, thus affecting the volume and texture of the final product. The appropriate viscosity of the dough maintains the air bubbles during mixing, thus increasing the specific volume of the product (Kim et al. 2017). Replacement of oleogel in the product by changing the viscosity of the mixture and the distribution of air bubbles in the dough affects density and the bakery and confectionery products structure (Pehlivanoglu et al. 2018). Figure 4c shows consuming water‐in‐oleogels increased the density of croissants by reducing the oil proportion and changing its quality. In samples prepared with water‐in‐oleogel containing hydrocolloid and Zein, this density change is noticeable. Among the samples, the croissant prepared with water‐in‐oleogel containing 2.5% Zein had the lowest density compared to the other samples, and no significant difference was observed in the sample prepared with shortening. Consuming appropriate gelator in organogel and replacing proper levels of organogel with oil in the product have significant effect on the quality of confectionery products. A decrease in the specific volume of muffins prepared by replacing shortening with oleogel containing hydroxypropyl methyl cellulose was reported by (Oh ImKyung and Lee SuYong 2018). Increasing the level of oleogel prepared from grape seed oil containing carnauba wax in low‐fat muffins led to an increase in the density of the samples, while the muffins produced at low levels showed a proper and identical quality vs. the control (Hanifi‐Vahed, Salehifar, and Rahman 2022). The type and concentration of the gelator consumed in the production of organogel have a significant effect on the quality changes of the product, and its proper selection is necessary.

#### 3.3.4 Hardness

Texture is an important property in bakery and confectionery products and an indicator for determining the product's shelf life (Pehlivanoglu et al. 2018). Textural changes in bakery and confectionery products are related to their density and porosity (Oh ImKyung and Lee SuYong 2018). Figure 4d shows that the hardness of all samples increases at a different rate during the storage. The phenomenon of moisture migration and its effect on starch retrogradation is the main reason for this observation (Kim et al. 2017). On the first day after production, no significant difference in hardness was observed between most of the samples prepared with water‐in‐oleogel and the sample prepared with shortening. On the 5th and 10th day after production, the sample prepared with water‐in‐oleogel containing 2.5% Zein had lower hardness compared to the sample prepared with shortening, and the samples prepared with water‐in‐oleogel containing 5% and 7.5% Zein showed a significant difference in texture hardness than the sample prepared with shortening. The increase in texture hardness of the samples prepared with oleogels containing Zein‐Persian gum had a significant difference compared to the other samples during the storage period. The change in the porosity and the textural properties of the bakery and confectionery products where the fat content has been replaced can be due to an excessive increase in the viscosity of the batter or the removal of the protective effect of saturated fatty acid crystals on the air bubbles in the texture (Hwang et al. 2018). In this context, the replacement of commercial shortening with carnauba wax‐canola oil oleogel in the cake indicates that the hardness of the product texture increases three fold by increasing the concentration of gelators in the replaced oleogels. A study (Kim et al. 2017) claimed that an increase in density and a decrease in porosity of cake in the presence of oleogel is the reason for the increase in hardness of product texture.

### 3.4 Color Measurement

**FIGURE 5:**
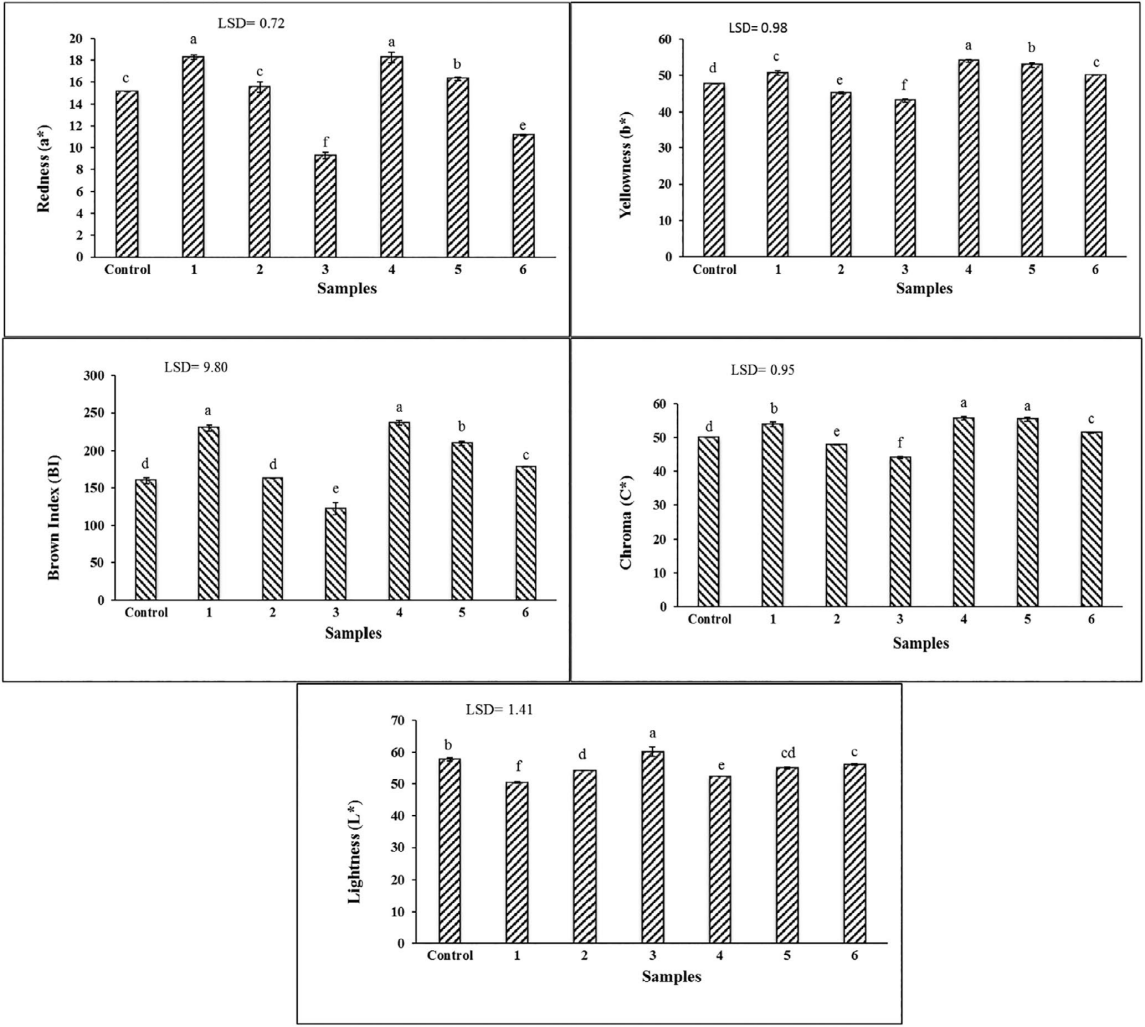
Mean comparison of color properties of croissants. Control is the croissant prepared with shortening. Croissants prepared with water‐in‐oleogel contain 1: (2.5% Zein), 2: (5% Zein), 3: (7.5% Zein), 4: (2.5% Zein and 1.5% Persian gum), 5: (5% Zein and 1.5% Persian gum), 6: (7.5% Zein and 1.5% Persian gum).

Changing the thermal properties of dough, like the heat and mass, transfer coefficient, and the effect of dough on quality, and moisture content of product, affect the surface color of bakery products (Goldstein and Seetharaman 2011). The brown crust is formed by a non‐enzymatic Maillard browning reaction between amino acids and reduced sugars (Wan‐Ibadullah et al. 2021). Increasing the color of the baked product's surface produced with pastry dough is an essential feature that contributes to consumer preference. Figure 5 shows that increasing the Zein concentration with and without hydrocolloid increased the brightness of the product (L*), and at low Zein concentration, the intensity of redness (a*), yellowness (b*), chroma (C*), and browning index (BI) of sample were higher than the sample prepared with shortening. Protein‐hydrocolloid interaction also increased b*, SI, and BI. Changes in the surface color of baked products due to organogel consumption have been reported in researches. Replacing shortening with wax‐based sunflower oil oleogels of rice bran wax, beeswax, and candela wax increased L* values in cake samples, while no significant difference was observed in a* and b* parameters (Amoah et al. 2017). Increasing the concentration of konjak, guar gums, and soy protein in the production of low‐fat and sugar muffins increases L* and decreases a* and b* of samples (Azmoon et al. 2021). The increase in oil crystallization by adding a gelator to the oleogel formulation and its effect on the reflection of radiant light is the reason for this observation (Oh ImKyung and Lee SuYong 2018).

### 3.5 Quality Properties of the Optimal and Control Sample

The croissant prepared with water‐in‐oleogel containing 2.5% Zein with the lowest fat release, density, and hardness and the highest similarity to the sample prepared with shortening is chosen as the better one.

**FIGURE 6:**
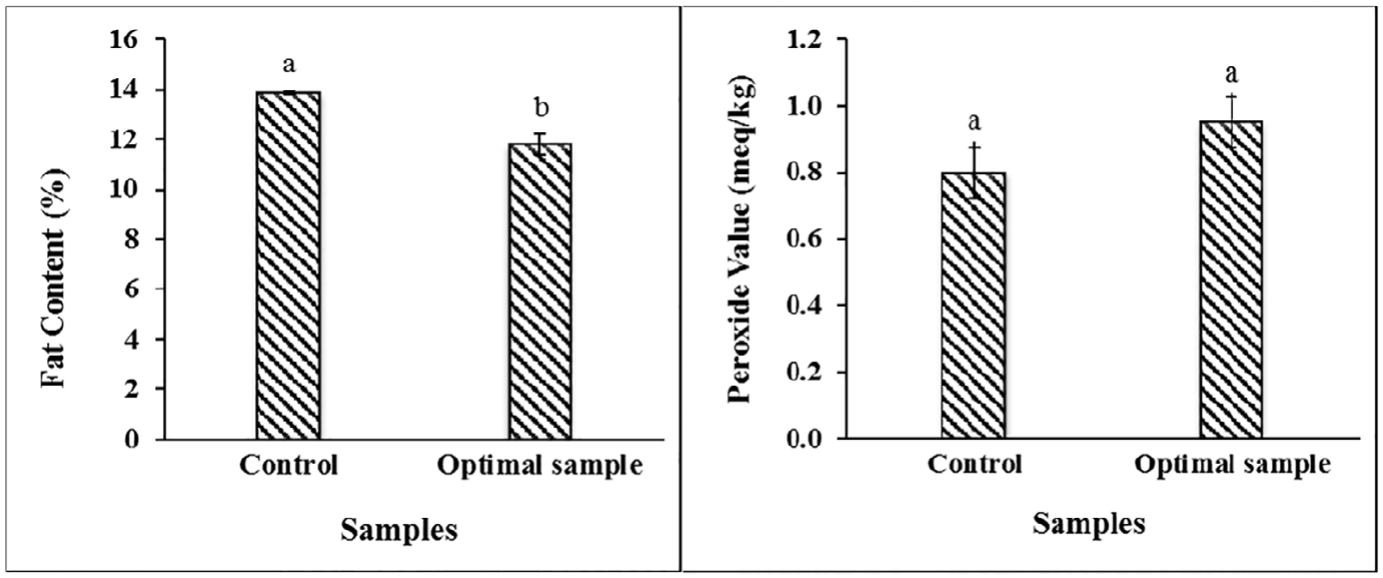
Mean comparison of fat content and peroxide value of samples. Control is the croissant prepared with shortening. The optimal sample is the croissant prepared with water‐in‐oleogel containing 2.5% Zein.

The results of the assessment and comparison of the fat content of the optimal sample and the sample prepared with shortening as the control in Figure 6a indicate that by replacing the water‐in‐oleogel in the croissant, next to preserving the qualitative properties of the product, significantly decreased its fat content by 15% compared to the sample prepared with shortening. Many studies have been run on reducing the fat content of bakery and confectionery products through different fat substitutes or production techniques. Assessing the impact of butterfat content and composition on the quality of laminated pastries revealed that samples with high fat were easier to manipulate and form and had high volume and soft texture, and high unsaturated fat content yields low volume and high density products (Ramirez 2020). In an evaluative study on corn maltodextrins and pectin as fat replacers in the production of low‐fat (low‐calorie) croissants, the results revealed that most of the organoleptic properties were adversely affected as a function of increasing levels of fat replacers and croissants containing pectin were harder than others (Shouk and El‐Faham 2005). The effect of the hydrocolloids–protein mixture, including konjac and guar gums and soy protein isolate, as a fat replacer in sugar‐free, low‐fat muffin cakes was assessed (Azmoon et al. 2021), where, though the increase in the mixture content increased the moisture content, specific volume, springiness, cohesiveness, and chewiness, it did not affect the product hardness. Consequently, it is possible to substitute water‐in‐oleogel with shortening in the croissant formulation while maintaining the qualitative properties of the product. In this case, in addition to reducing the fat content of the product, the percentage of saturated fatty acids is reduced.

According to the results in Figure 6b, the sample prepared with water‐in‐oleogel and shortening had no significant difference in terms of peroxide value, and its quantity was within the appropriate range of Standard 2553. Although sunflower oil with high levels of unsaturated fatty acids has been consumed in the water‐in‐oleogel formulation, no excess oxidation is observed in the product during the cooking process. The formation of a hard, semi‐solid oleogel network protects the trapped oil against the oxidation reaction (Kim et al. 2017). According to Yılmaz and Öğütcü 2014, the volume of peroxide value of the samples containing sunflower wax and beeswax organogel compared to shortening after applying the heat process and storage at ambient and refrigerated temperatures was within the permissible range, and the organogels were structurally more stable during the storage period. Researchers (Silva et al. 2021) found that the organogelation process has no effect on the destruction of antioxidant compounds in olive oil but has been contributing to preventing the oxidation of this oil by increasing the content of soluble solid compounds like waxes. The main difference between oil types is in their fatty acids' composition and the different properties of organogels and shortenings made with different oil types which is attributed to the difference in the profile of fatty acids (Hwang et al. 2018). According to Table 3, the total volume of saturated fatty acids in the sample prepared with water‐in‐oleogel, compared to the sample prepared with shortening, decreased by 47%, and the essential unsaturated fatty acids increased by 65%. In this study, a 3‐fold increase was observed in linoleic acid (the main fatty acid of sunflower oil) in croissants prepared with water‐in‐oleogel. The highest percentage of fatty acid in the sample prepared with shortening was palmitic acid. Trans‐elaidic fatty acid (18:1 Trans) was present in croissants prepared with shortening in a high proportion. Replacing fat with oleogel in the muffin formulation led to a 68% decrease in saturated fatty acids and a 4‐fold increase in mono‐unsaturated fatty acids (Giacomozzi, Carrín, and Palla 2023). The level of saturated fatty acids in ice cream produced with linseed oil organogel with rice bran wax decreased and the level of unsaturated fatty acids increased significantly compared to the control (Banupriya et al. 2016). Considering the effect of saturated fatty acids on cardiovascular diseases, increasing obesity, and diabetes, it is essential to reduce the hydrogenated fats content in food products if the high quality of the product is sought (Anushree et al. 2017).

**TABLE 3:** Fatty acids profile of croissant samples.

Fatty acids	Amount of fatty acids (%)	
	Optimal sample	Control sample
Myristic acid (C_14:0_)	—	1.44 **±** 0.04
Palmitic acid (C_16:0_)	20.05 **±** 0.50	38.69 **±** 0.74
Stearic acid (C_18:0_)	10.50 **±** 0.11	17.91 **±** 0.03
Oleic acid (C_18:1 cis_)	20.06 **±** 0.70	25.68 **±** 0.89
Elaidic acid (C_18:1 trans_)	—	0.70 **±** 0.07
Linoleic acid (C_18:2 cis_)	36.49 **±** 0.72	13.36 **±** 0.32
Linolenic acid (C_18:3_)	—	0.55 **±** 0.01
Total saturated fatty acids	30.55 **±** 0.44	58.04 **±** 0.65
Total unsaturated fatty acids	69.42 **±** 0.97	41.96 **±** 0.98

According to Table 4, there exists no significant difference in organoleptic properties like aroma, taste, color, texture, and overall acceptance between the sample prepared with water‐in‐oleogel and the sample prepared with shortening at (*p* > 0.005). Therefore, by concentrating on the organoleptic properties, it is possible to substitute appropriate water‐in‐oleogel with shortening in the croissant formulation for reducing the fat content and the saturated fatty acids of the product. Similar results were reported in studies run on other bakery and confectionery products. The cake samples prepared with oleogel containing carnauba wax had similar organoleptic properties to those available in the control (Pehlivanoglu et al. 2018). Researchers (Espert et al. 2023) reported that although increasing the concentration of oleogel containing HPMC in the croissant increased the consistency and chewing ability of the samples, certain and appropriate concentrations in the formulation produced the most similar organoleptic properties to the prepared sample with hydrogenated fat.

**TABLE 4:** Results of Mann–Whitney *U* non‐parametric test to compare the average of sensory data.

Samples	Texture	Taste	Aroma	Color	Overall acceptance
Control	4.30 **±** 0.73^a^	3.30 **±** 1.03^a^	3.65 **±** 0.099^a^	4.20 **±** 0.89^a^	4.00 **±** 0.92^a^
Optimal	3.85 **±** 0.67^ab^	3.55 **±** 0.95^a^	3.20 **±** 0.95^a^	4.00 **±** 0.86^a^	3.80 **±** 0.77^a^

*Note:* In each column, means (±standard deviation) that have different letters have significant differences. Control is the croissant sample prepared with shortening.

## 4 Conclusions

In this study, to assess the mutual effects of different gelators, a combination of Zein protein, Persian gum, and glycerol monostearate was consumed to produce water‐in‐oleogels from sunflower oil. The presence of Persian gum in the gelator slightly increased the melting point of water‐in‐oleogels. The shear thinning behavior with a higher consistency coefficient in the presence of hydrocolloids is revealed here. The results of replacing shortening with oleogels in the croissant formulation indicate that the presence of Persian gum and the increase in Zein concentration in the water‐in‐oleogels improved the preservation of moisture content of the product. No significant difference was observed in hardness among most of the samples prepared with water‐in‐oleogels and the sample with shortening on the first day after production. Assessing the texture of the samples at the 5th and the 10th days after production revealed that the samples prepared with water‐in‐oleogels containing 5% and 7.5% Zein did not have a significant difference in terms of texture and hardness with the sample prepared with shortening. Croissants produced with water‐in‐oleogel containing 2.5% Zein had a better texture compared to the sample prepared with shortening. Increasing the Zein concentration in the presence of hydrocolloid enhanced the lightness of the product (L*). Considering all quality parameters, the croissant prepared with oleogel containing 2.5% Zein was selected as the optimal sample. The optimal croissant produced with water‐in‐oleogel contained 15% less fat compared to the sample produced with commercial shortening, and no significant difference was observed in terms of peroxide value of them. The total saturated fatty acids in the sample prepared with water‐in‐oleogel decreased by 47%, and the essential unsaturated fatty acids increased by 65% compared to the sample prepared with shortening. There was no significant difference between these two samples in terms of most organoleptic properties, including aroma, taste, color, and general acceptance. Consequently, the produced novel bigels had suitable rheological and thermal properties and can be used in the formulation of food products depending on the desired characteristics of fat in them.

## Ethics Statement

This article does not contain any studies concerned with human participants or animals.

## Conflicts of Interest

The authors declare no conflicts of interest.

## Data Availability

The data that support the findings of this study are available on request from the corresponding author.
